# Pollution Assessment of Potentially Toxic Elements (PTEs) in Soils around the Yanzhuang Gold Mine Tailings Pond, Pinggu County, Beijing, China

**DOI:** 10.3390/ijerph18147240

**Published:** 2021-07-06

**Authors:** Guangjie Zhao, Xianqing Li, Jiewang Zhu, Xueyan Zhao, Jizhen Zhang, Jia Zhai

**Affiliations:** 1 State Key Laboratory of Coal Resources and Safe Mining, China University of Mining and Technology, Beijing 100083, China; zhaoguangjie23@126.com (G.Z.); zhaijiachem@163.com (J.Z.); 2 College of Geoscience Grad Surveying Engineering, China University of Technology, Beijing 100083, China; 3 School of Civil Engineering, Shandong Jianzhu University, Jinan 250101, China; 4 Chinese Research Academy of Environmental Sciences, Beijing 100012, China; zhaoxy@craes.org.cn; 5 Key Laboratory of Exploration Technologies for Oil and Gas Resources, Yangtze University, Ministry of Education, Wuhan 430100, China; zhangjz1991@126.com; 6 College of Resources and Environment, Yangtze University, Wuhan 430100, China

**Keywords:** potentially toxic elements, environmental pollution assessment, tailings pond, geoaccumulation index, enrichment factor, potential ecological risk

## Abstract

The accumulation of tailings from gold mining and smelting may result in PTE pollution. We investigated PTE contamination from a large amalgamated gold mine tailings pond in Pinggu County, Beijing. In November 2017, 30 soil samples were collected around the tailings pond. The concentrations and pollution degree of PTEs in the samples and the sources of Sb, As, Cd, Cu, Pb, Zn and Hg were analyzed. The average concentration of these elements in soil samples near the tailings pond (16.24, 28.29, 0.99, 171.04, 263.25, 99.73, 0.72 mg/kg, respectively) were higher than their corresponding standard values and background values of the study area. The geoaccumulation index showed that the pollution degree of As, Pb and Hg was moderate, while Sb and Cu present non-pollution to moderate pollution. The average EF values of the elements were Sb (38.31), As (4.23), Cd (0.71), Cu (3.68), Pb (21.24), Zn (0.82) and Hg (5.29), respectively. The environmental risk assessment developed throughout the PERI method indicated that Sb, As, Hg and Pb were the main pollutants in the study area. The three quantitative risk indicators (RI, Igeo and EF) were positively correlated, and all of them indicated that PTEs had significant pollution to the local area. Thus, Sb, As, Pb, Cu, and Hg pollution should be highly concerning. Multivariate statistical analysis shows that the pollution of PTEs was mainly caused by the accumulation of tailings ponds after gold mining and smelting. The research result is of great significance for the prevention and control of soil pollution of PTEs near the tailings pond.

## 1. Introduction

Potentially Toxic Elements pollution in tailing ponds has an increasingly serious impact on the environment, which has attracted global attention [1,2,3,4,5]. The mobility of PTEs (i.e., Cu, Pb, Zn, Cd) mainly determines the environmental hazards of PTEs in tailings [6]. Furthermore, large amounts of huge tailings ponds that have not been treated for a long time are accumulated in large quantities and subsequently exposed to natural conditions such as surface water runoff, infiltration and weathering. The discharge of PTEs with high toxicity (i.e., As, Cd) can pollute the soil and pose health risks to people nearby through different channels [7,8]. As a result of high PTE concentration, the nearby residents got various health problems [9,10]. Worse still, a large number of PTEs from mining activities and tailings have been transferred to agricultural soil, which may pollute agricultural products [11,12,13]. Due to the above facts, soil environmental sampling has become a crucial means to evaluate the anthropogenic pollution with PTEs. Over the past few years, several studies to determine the contamination of PTEs in the soils of neighboring gold mines have been conducted in many countries [14,15,16,17,18].

The relationship between Hg contamination and artisanal and small-scale gold mining (ASGM) technology is direct [19]. The amalgamation method is a gold extraction method, which is commonly found in Tanzania, Ecuador, Brazil, China [20,21,22] and other countries. The waste of ASGM was discharged to land without any treatment, which will pollute the surrounding soil. In China, it was found that the concentration of Hg in tailings was relatively higher than background concentrations [14]. Moreover, in Indonesia, ASGM activities in Central Java caused a great deal of Hg pollution to local streams [23]. In the small-scale gold mining in Bolivar (Colombia), soils surrounding mining industry were also seriously contaminated by Hg [24]. In 1996, the cyanidation method replaced the amalgamation method in the gold smelting process and was frequently used in China. Although there is no mercury added in the cyanidation process, this method also produces different pollution [25].

In 2005, the Pinggu County Government closed the tailing pond of Yanzhuang Village. The amalgamation method and cyanidation method were mainly used in the ore for beneficiation. The tailings pond seriously and continuously affects the surrounding environment. According to local villagers’ responses, in the rainy season, poultry and livestock sometimes died from drinking water near the tailings pond. The reason may be the migration and transformation of trace element pollutants, which bring potential risks to the environment [26]. In addition, the lack of scientific data on the distribution and development of pollutants has severely restricted the mitigation and remediation of water pollution in the region. Hence, the PTEs pollution in the soil of this gold mine tailing pond was investigated in order to obtain information and take reasonable measures to reduce pollution in time.

The purposes of this study are: (1) to measure the concentration of PTEs, (2) to study the spatial distribution characteristics of PTEs in tailings pond pollution, (3) to evaluate the potential sources and ecological risk of the PTEs. This result is of great significance for pollution prevention and control of gold tailings ponds in Beijing.

## 2. Materials and Methods

### 2.1. Study Area

**Figure 1:**
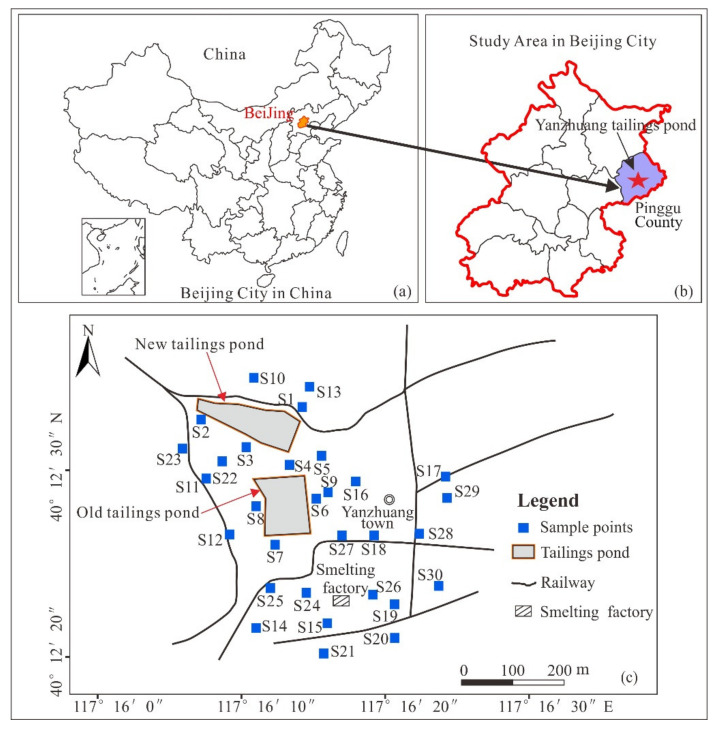
(**a**) Geographic location of China. (**b**) The location of the study area in Beijing. (**c**) Schematic map of the studied area and sampling points.

The study area is about 0.1 km^2^, which is located in the east of Pinggu County, Beijing, China (Figure 1). Beijing, located in northern China, is the most important city in the region. The distance from the research area to the center of Beijing is about 70 km. The climate in the area is characterized by temperate continental monsoon climate. It is hot and rainy in summer and cool and humid in autumn. The annual average temperature is less than 10 °C, and the annual precipitation is 600–700 mm. Precipitation is unevenly distributed throughout the year, with more than 80% of the rainfall concentrated in June to September. The Yanzhuang gold mining area lies from the latitudes 40°12′20″ N to 40°12′40″ N and from the longitudes 117°16′00″ E to 117°16′25″ E. Miyun Reservoir, located in the north of Yanzhuang Tailings Pond, is an important drinking water source and ecological protection development zone of the capital. And it may be affected by the seepage pollution of tailings pond. There are hundreds of villagers around the tailings pond, whose crops and drinking water could be affected by the tailings.

According to the data and understanding on the scene, Yanzhuang (old) tailings pond has existed before and after the founding of the People’s Republic of China, but tailings began to be piled up in Yanzhuang (new) tailings ponds in the 1990 s. Before 2005, the tailing sands of the two tailings ponds were both scattered in the nearby area and no reservoir was built for closure. In 2005, Pinggu County government closed the tailing ponds in Pinggu County uniformly. Afterwards, tailings dam was reinforced, forming the current tailings dam. Yanzhuang (old) tailings pond and Yanzhuang (new) tailings pond were 50 m apart from each other, and they were located in the same topographic environment, which had an interactive effect on the pollution nearby. Therefore, during the environmental investigation, the layout and samples of the two tailings ponds were carried out together, and the subsequent pollution analysis was also conducted on the surrounding environment of the two tailings ponds. This research mainly focuses on the agricultural land around the tailings pond. The tailings pond area primarily consists of quaternary sediments, and brown soil and waterlogged soil were formed from them.

### 2.2. Sample Collection

In November 2017, a randomized method was employed to form a sampling network near the tailings pond (Figure 1), according to the Technical Specification for Soil Environmental Monitoring (HJ/T 166-2004). Each sample consisted of four homogeneous subsamples located in an imaginary circle about 4 m in diameter around the sampling site. A total of 30 topsoil samples were collected with a sampling depth of 0–20 cm. A Geographical Position System (GPS) device was used to record the geographical coordinates of the sampling sites in detail. All collected samples were packed in polyethylene bags and labeled. The bags were sealed and transported to the lab for further treatment as soon as possible. The “Background Concentration of Soil Elements in China” (1990 China National Environmental Monitoring Center) report provided the background concentration of PTEs in this study.

### 2.3. Methods

#### 2.3.1. Analysis Methods

All the samples were naturally air-dried at room temperature in the laboratory. The moisture content of soil samples ranged from 3.5% to 5.5%. Afterward, they were divided and then were passed through a sieve with a diameter of 0.15 mm. Since they need to be treated, this includes completely removing unwanted impurities (stones, plant debris, etc.) and crushing them into a fine powder. Clean polyethylene bags were used to store soil powder. The samples were digested by multivariate mixed acid digestion method (HCl-HNO_3_-HF-HClO_4_). Before almost evaporating to dryness and no more white fuming, the extract was poured into a glass tube after cooling, and then diluted with 2% ultrapure HNO_3_ to 50 mL, and then filtered with a 0.45 μm membrane filter for analysis. The contents of Sb, Cd, Cu, Pb and Zn were measured by atomic absorption spectroscopy (AA-300; PerkinElmer Co., Waltham, MA, USA), and As and Hg contents were quantitatively tested by atomic fluorescence spectroscopy (AFS920; Beijing Jitian Instrument Co., Beijing, China). In order to ensure the precision and accuracy of the experimental results, standard reference materials (SRM) were used to determine the quality of each method. The recovery efficiency of the elements in the standard soil sample (GBW07427) varied from 92.3% to 104.8%. The measurement was repeated 3 times for each sample, with the relative standard deviation (RSD) less than 3%, complying with the provisions of the technical specifications for soil environmental monitoring.

#### 2.3.2. Statistical Analysis

The analytical data were processed and analyzed using Microsoft Excel 2007, IBM SPSS Statistics 22.0, Origin Pro 9.0. The relationship between elements and their possible sources is analyzed by principal component analysis (PCA), Pearson correlation coefficient analysis, and cluster analysis (CA). The relationship between different PTEs can be analyzed by the PCA method [27]. Correlation coefficient analysis [28] is also utilized to identify the relationship among PTEs and their possible sources. CA [29] can be used to further classify the similarity of soil sample attributes.

#### 2.3.3. Geochemical Accumulation Index

The impact of human activities on the environment will cause changes in the content of PTEs. Generally speaking, the geochemical accumulation index (Igeo) method is used to study the degree of PTEs pollution in sediments. This index was designed by Müller [30] using the formula:

(1) $$I_{\mathrm{geo}}=\log_{2}\frac{\mathrm{Ci}}{k\times \mathrm{Bi}}$$

In the formula, Ci is the measured content of element (i) in the soil, Bi is the geochemical background value of element (i) in the soil, and k is the background correction factor due to lithological movement (the value is 1.5) [31]. According to the results, the seven different categories were classified as follows: <0 (Non-pollution), 0–1 (Non-pollution to moderate pollution), 1–2 (Moderate pollution), 2–3 (Moderate to strong pollution), 3–4 (Strong pollution), 4–5 (Strong to extremely pollution) and >5 (Extremely pollution).

#### 2.3.4. Enrichment Factor

The enrichment factor (EF) can be used to determine whether the elements in the soil are from nature or are affected by anthropogenic factors. The method used is to normalize the measured element against the corresponding reference element to identify the degree of influence of anthropogenic factors on the target environmental medium. The selected reference elements are usually inert elements with weak volatility and high chemical stability, such as Fe, Al, Sc or Ti [32,33]. The calculation formula of EF is as follows:

(2) $$\mathrm{EF}=\left[\frac{{\left(\mathrm{Xi}\right)}_{\mathrm{sample}}{/(\mathrm{RE})}_{\mathrm{sample}}}{(\mathrm{Xi}{)}_{\mathrm{crust}}/(\mathrm{RE}{)}_{\mathrm{crust}}}\right]$$

where EF is the enrichment factor, (Xi)_sample_ is the measured concentration of the element, (RE)_sample_ is the measured concentration of the reference element in soils in the study area, (Xi)_crust_ is the average concentration of the element in the crust, (RE)_crust_ is the average concentration of reference elements. In the current research, since the Sc element mainly occurs naturally, according to the “Background Value of Soil Elements in China” (SEPAC 1990), Sc (11.26 mg/kg) is used as the reference element in the formula. The abundances of Sb, As, Cd, Cu, Pb, Zn, Hg and Sc in the earth’s crust have been calculated by the previous scholar to be 0.2, 1.8, 0.2, 55, 12.5, 70, 0.08 and 22 mg/kg, respectively [34]. According to the EF value, five contamination categories are defined [35], as shown in Table 1.

#### 2.3.5. Potential Ecological Risk Index

In order to determine the status and extent of PTEs in soil, a large number of standard indicators, including the Nemerow composite index, geological accumulation index, enrichment factor and potential ecological risk index, need to be calculated [26,36,37,38]. However, among these methods, the potential ecological risk index (PERI) method has been widely used by many scholars to quantitatively assess the negative environmental impact and comprehensive potential ecological risk caused by toxic elements [26]. The core of potential risk index evaluation is potential risk index (RI). Based on the rule of element abundance, it classifies the potential harm degree of PTEs by quantitative method, which can combine the pollutant concentration with the biological toxicity and ecological harm organically. The impact potential of PTEs on ecological environment is comprehensively reflected [39].

The mathematical expression of the potential ecological risk index is as follows:

(3) $${E_{r}}^{i}={T_{r}}^{i}\times {C_{f}}^{i}$$

(4) $${C_{f}}^{i}={C_{D}}^{i}/{C_{n}}^{i}$$

In Equation (3), T_r_^i^ is the toxic response factor; C_f_^i^ is the factor of contamination. T_r_^i^ value is the toxicity response factor of standardized PTEs formulated by Håkanson as the evaluation basis, and their toxicity response coefficients (Sb, As, Cd, Cu, Pb, Zn and Hg) are 10,10, 30, 5, 5, 1 and 40, respectively. E_r_^i^ is the potential ecological risk factor. In Equation (4), C_D_^i^ is the content of PTEs in surface soil, and C_n_^i^ is the parameter ratio of PTEs content in soil. In this paper, the background values measured from the samples were taken as the parameter ratio.

RI (the potential ecological risk index) is shown in the following formula.

(5) $$\mathrm{RI}=\sum_{i=1}^{n}{E_{r}}^{i}$$

According to Håkanson’s grading principle for RI, the classification criteria for the potential risk coefficient and potential ecological hazard index of PTEs are shown in Table 2.

## 3. Results and Discussion

### 3.1. Basic Analysis of PTEs Concentrations

The descriptive statistics of the content of potentially toxic elements in the soil samples near the gold mine tailing pond were summarized in Table 3, which also included the Chinese soil quality standards and the background soil values in the study area for evaluation and comparison.

The result showed that the concentrations obtained at different sampling points were significantly different. It could be seen from Table 3 that the average contents of Pb and Zn in the soil were lower than the acceptable threshold of the Grade II of China Soil Environmental Quality Standard (GB15618-1995) [40]. Compared with the background values of Beijing soil, except for Zn, the average concentrations of other elements Sb, As, Cd, Cu, Pb and Hg were significantly higher. It should be pointed out that the contents of Sb, Cd, Pb and Hg were about 15.04 times, 18.54 times, 10.66 and 12.51 times of the corresponding background values, respectively. The above results revealed that the content of Sb, Cd, Pb and Hg near the tailings pond exceeded the standard in recent decades, which may be closely related to the mining and accumulation of tailings ponds, and its potential pollution risk was worthy of attention. The distribution of the mean values and standard deviation of the detection data is abnormal, which corresponds to the conclusion of these articles [9,41,42].

The coefficient of variation reflects the distribution of the element in the soil. The larger the coefficient value, the more uneven the spatial distribution of the element contents and the greater the interference from human activities [43]. The variation coefficients for the elements (CV = SD/mean 100) followed the order As (56.52%) < Sb (74.32%) < Zn (90.25%) < Cu (101.34%) < Pb (132.54%) < Cd (142.42%) < Hg (187.51%), which demonstrated that the variability of these measurements raised under a large concentration variation (CV > 36%) [44].

### 3.2. Spatial Distributions of PTEs

**Figure 2:**
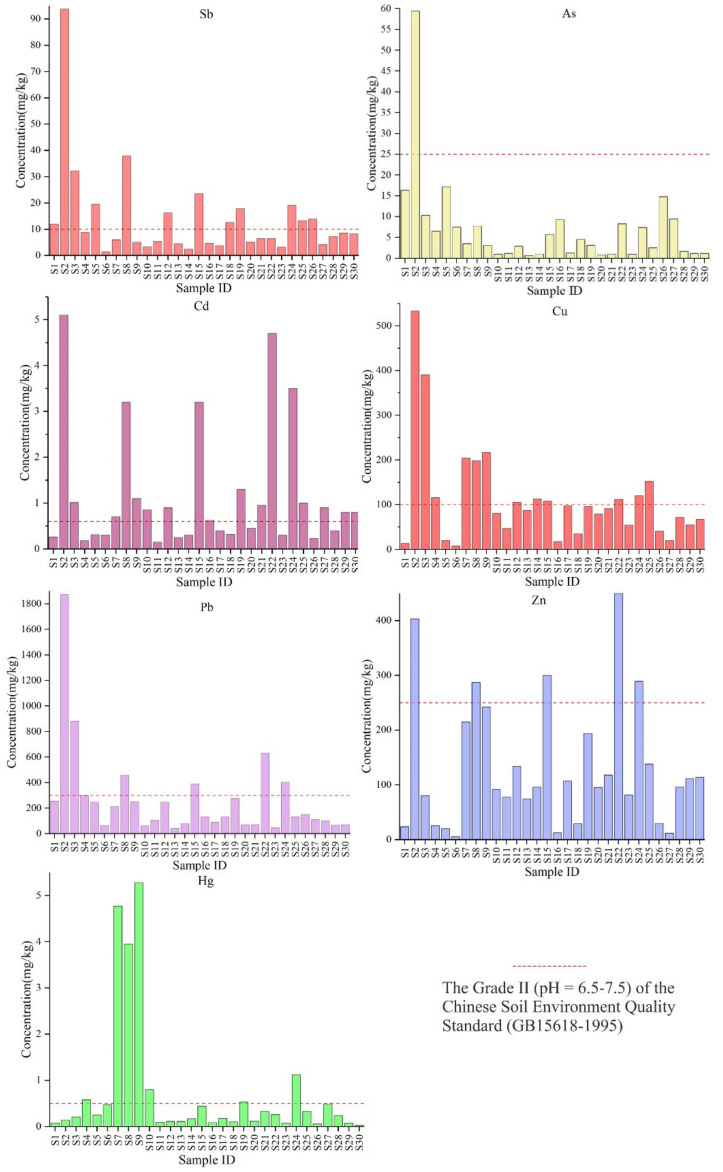
Spatial distribution of the studied PTES concentrations in soils (The critical values from the Chinese Soil Environment Quality Standard (GB15618-1995) were marked for all PTEs).

The spatial distribution trends of the investigated PTEs in the soil samples near the tailings pond is shown in Figure 2.

Sb, As, Cu and Pb have significantly similar spatial distribution trends. In addition, there were relatively high concentrations near the smelting plant in the southeast of the tailings pond. What is interesting is that among the 30 sampling points, the maximum concentration was found near this point (S2). The distribution characteristics may be related to the fact that point S2 is very close to the tailings pond. And their concentration distribution trends show a downward trend in similar locations. For Cd and Zn, they have no obvious spatial distribution trend, and there is no regularity, which is different from the above four elements. The trend of Hg concentration distribution is obviously different from other PTEs, whose peak concentration of the sample is between S7–S9. It is worth noting that the highest concentration of Hg at sampling point S9 is 91.67 times that of the background value of the study area.

The spatial distribution pattern may be due to mining, smelting and other activities and the accumulation of tailings reservoirs that seep into the soil and spread around under the erosion of water, which is the result of a comprehensive effect. Typically, the high concentration distribution trend of PTEs can be explained by a large number of mining and smelting activities.

### 3.3. Geoaccumulation Index and Enrichment Factor

**Figure 3:**
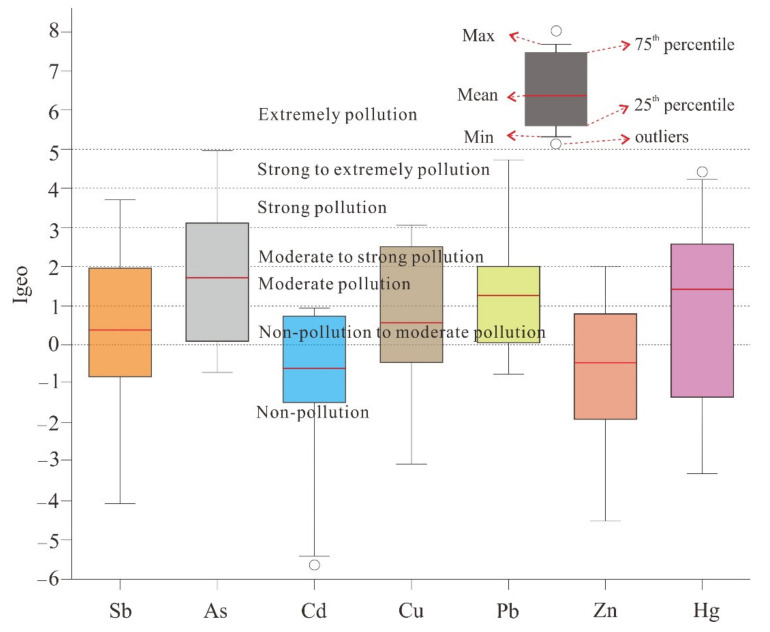
Whisker-box plots for geoaccumulation index of PTEs in soils.

The geochemical accumulation index (Igeo) was designed to evaluate the contamination level of PTEs (Figure 3). The order of the mean Igeo value of PTEs is As (1.71 ± 1.56) > Hg (1.41 ± 1.84) > Pb (1.31 ± 1.12) > Cu (1.23 ± 1.68) > Sb (1.21 ± 2.45) > Zn (−0.4 ± 1.59) > Cd (−0.5 ± 2.81). Therefore, combining each potential toxic element and the location of the sampling station, Igeo indicated non-pollution to strong pollution of the PTEs in the study area. Then, As, Pb and Hg present a moderate pollution (1 < Igeo < 2), while Sb and Cu present non-pollution to moderate pollution (0 < Igeo < 1). Again, the average Igeo values of Cd and Zn elements are less than 0, which indicates non-pollution (Igeo < 0).

**Figure 4:**
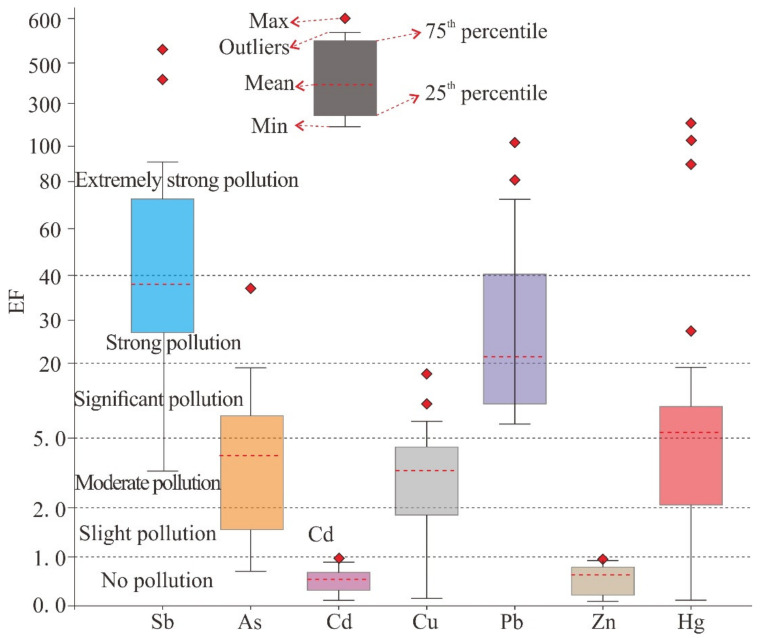
Whisker-box plots for enrichment factors (EFs) and pollution grades of the studied PTEs in soils.

The EFs are shown in Figure 4 to demonstrate their variability between different locations in the study area. The calculation results of enrichment factors indicate that Sb, As, Cu, Pb and Hg (EF values are 38.31, 4.23, 3.68, 21.24, 5.29, respectively) are enriched in the soils near the tailings pond [35], which should get more attention in the future. The result of EF value analysis is similar to that of Igeo. However, some differences exist between the Igeo results and the EF results. The former revealed that Sb is classified as non-pollution to moderate pollution, while the latter as extremely strong pollution. The results may be related to the content of element and the principle of pollution level. Sb had the highest EF values among the seven PTEs and it was enriched with strong pollution level (average value 38.31). The average EF values reveal slight enrichment for Cd and Zn (average value 0.71 and 0.82, respectively), with EF values < 1.0 and had no enrichment. Moreover, the relationship between elements in soil samples provides information on the sources and pathways of PTEs in the earth’s environment [46].

### 3.4. Multivariate Statistics for Pollution Source Apportionment

#### 3.4.1. Pearson’s Correlation Analysis of PTEs

Pearson Correlation Matrix (PCM), a multivariate analysis technique, is used to identify the potential sources of PTEs and assess the degree of their quantitative correlation [47]. PTEs with the same origin or similar geochemical activities [37] may cause significant correlation between them. The results related to the PTEs in the soil samples surrounding the tailings pond are shown in Table 4. Generally, the significant positive correlation between the concentrations of PTEs can indicate they derive from similar sources. A significant strong positive correlation is present between Sb and As (r = 0.913, *p* < 0.01), Sb and Cu (r = 0.813, *p* < 0.01), Sb and Pb (r = 0.825, *p* < 0.01), As and Cu (r = 0.827, *p* < 0.01). And a significant moderate positive correlation is present between As and Pb (r = 0.542, *p* < 0.05) and Cu and Pb (r = 0.461, *p* < 0.05). Furthermore, a significant strong positive correlation is present between Cd and Zn (r = 0.786, *p* < 0.01), but the two elements do not show significant positive correlation with the above elements, which suggests they may have different origins. These significant and strong correlations indicate that these PTEs in the soils around the Yanzhuang tailings pond originate from similar sources, which are mainly from human activities. However, Hg is not positively correlated with all of the rest of the PTEs, indicating that Hg may have a completely different source from other elements. As mentioned above, the enrichment level of Hg is relatively high. This may be because workers used amalgamation method for gold smelting, so a lot of Hg was added.

#### 3.4.2. Principal Component Analysis

**Figure 5:**
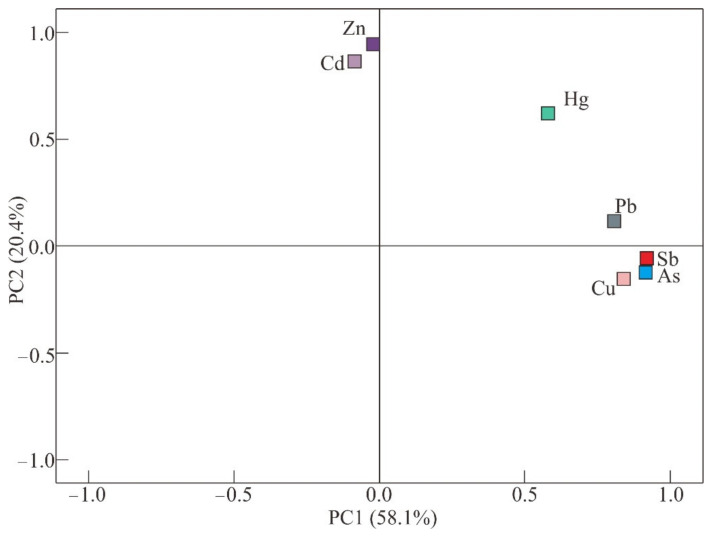
Factor loadings of two principle components after varimax rotation.

PCA is used to further evaluate the pollution degree of PTEs in the study area and identify the source. The content of PTEs and the results of principal component analysis at sampling points are shown in Table 4 and Figure 5. The results show that there exist two eigenvalues greater than 1, these two factors accounted for 78.5% of the total variance. Sb, As, Cu and Pb are contained in the first component (PC1), while the second component (PC2) contains Cd and Zn. Hg is an element that is not clearly associated with the first or second component, and PC1 (0.621) has a similar moderate loading value compared with PC2 (0.594).

In detail, PC1, which presents high loading values for Sb, As, Cu and Pb, explains 58.1% of the variance and is the most significant component. PC1 could be reasonably interpreted as the reason of anthropogenic activity, especially originate from gold mining. It was reported that main minerals in ore were native gold, galena, chalcopyrite, molybdenite, senarmontite, etc. [48]. Previous scholars revealed that As, Pb and Cu were relatively enriched in ore [49]. Sb, As, Cu and Pb might be released from minerals related to gold deposits. The above rationales indicate that PC1 can be considered as an anthropogenic component.

PC2 accounts for 20.4% of the total variability and can be presumed to natural source. Based on the results of correlation analysis, there is a significant positive correlation between Cd and Zn, but they are not correlated with Sb, As, Cu and Pb. According to Igeo and EF results, they are not contaminated, but their concentrations are higher than the corresponding background values. So, it suggests that PC2 are probably mainly natural sources, from geochemical activities or parent material of soil.

In addition, the presence of Hg in PC1 and PC2 (the high loading is 0.621 and 0.594, respectively), indicates that Hg may have a mixed source of geochemical activities and human activities at the same time [36].

#### 3.4.3. Cluster Analysis Results

**Figure 6:**
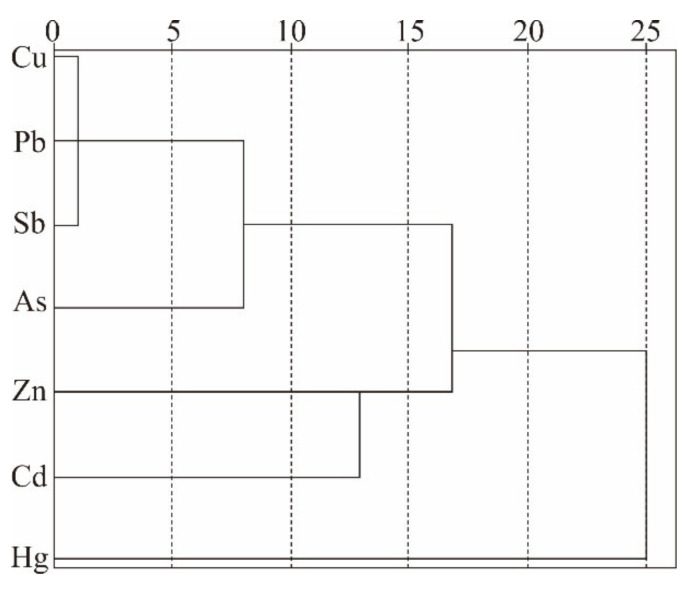
Hierarchical diagrams for the PTEs in obtained soils by the cluster analysis method.

The clustering method obtains different classes or clusters through data classification. In this paper, stratified clustering is selected, and tree diagram is used to vividly reflect the similarity or affinity relationship between PTEs in soils, so as to better understand the source of pollutants. Cluster analysis of 7 kinds of PTEs in the soil of gold mining area was carried out in Figure 6. According to cluster analysis, these seven elements were classified into three categories. The first category included Sb, As, Cu and Pb. The second category included Cd and Zn. And the third category included Hg. The results roughly corresponded to the spatial distribution characteristics, Pearson correlation analysis, and PCA.

### 3.5. Analysis of the Potentially Ecological Risk Assessment

**Figure 7:**
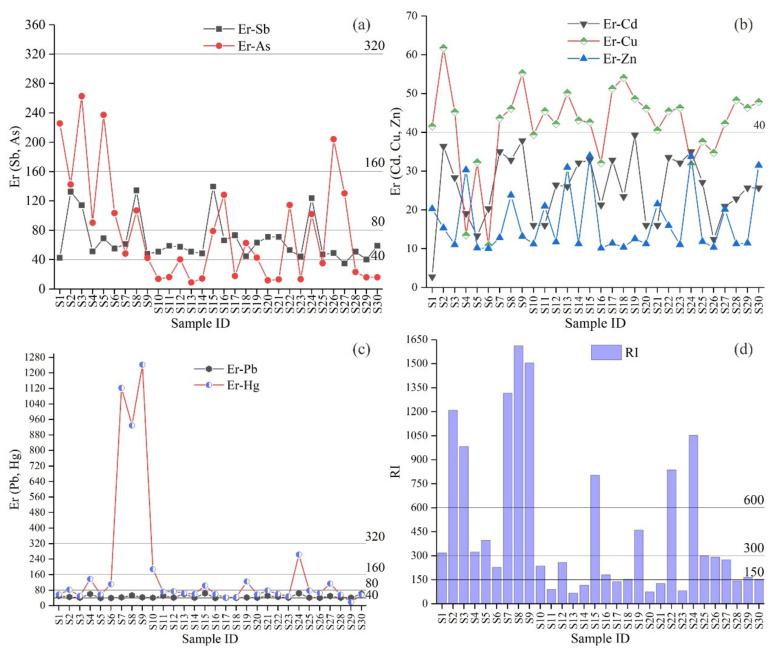
(**a**–**c**). Ecological risk (Er) and (**d**) Risk Index (RI) values of the PTEs in the study area.

In the present study, the Håkanson method is used to calculate the potential ecological risk index (PERI) of potentially toxic elements. The results of individual elements (Er) and multiple elements (RI) are presented in Table 5 and Figure 7. The mean Er values for Sb, As, Cd, Cu, Pb, Zn and Hg are 66.18, 61.65, 25.86, 43.55, 47.43, 17.43 and 205.60, respectively. The Er values of Zn and Cd are both less than 40, which demonstrates that the ecological risks of these PTEs are slight.

The Er value of Sb is between 34.72 and 139.72, and its mean value is 66.18, which shows moderate to significant pollution. The Er value of As is between 8.97 and 262.76, and its mean value is 61.65, which shows slight to strong pollution. In addition, about 50% of the samples present moderate to significant pollution (Figure 7a). The Er value of Cu is between 10.93 and 61.76, and its mean value is 43.55, and about 70% of the values of the samples is more than 40, indicating it meanly shows moderate pollution (Figure 7b). The Er Pb range from 40.07 to 64.01, and the mean value is 47.43, indicating moderate ecological pollution. Furthermore, most of the value of Pb shows moderate ecological pollution. At last, the Er value of Hg is between 18.71 and 1242.35, and its mean value is 205.60, and about 50% of the values of the samples is more than 80, indicating it meanly shows strong pollution (Figure 7c). In summary, the ecological pollution levels from low to high is Zn, Cd, Cu, Sb, Pb, As and Hg, which are basically consistent with the conclusion of the article (only consider the order of three elements: Cd, Pb, and Hg) [36]. However, certain differences exist between the ecological risk results and the geoaccumulation index results and the enrichment factor results. The geoaccumulation index result shows that the As pollution in the study area is serious, the enrichment factor result shows that Sb and Pb is serious, while the ecological risk index result indicates that the ecological risk of Hg is higher than that of all other elements. It is two reasons that caused this result, which are the concentration of PTEs and their toxicity. Sb, As and Pb are the main pollutants with high concentration in the study area. On the contrary, despite the low concentration of Hg, it is highly toxic to humans and wildlife [50]. Therefore, the problem of Hg, Sb, As and Pb pollution in the study area is worthy of attention.

The results in Figure 7d and Table 5 indicate that the risk index (RI) of all samples ranges from 67.29 to 1612.55. According to the average value of RI (333.53), the samples have a high potential risk (300 ≤ RI < 600). The RI values of sites 2, 3, 7, 8, 9, 15, 22 and 24 are greater than 600, indicating that these sites have serious potential ecological risks. Then, the RI values of sites 1, 4, 5, 19 and 25 are between 300 and 600, indicating that these sites have high potential ecological risks. In general, potential ecological risk caused by the elements is at a considerable-risk status. The results show that Hg, As and Pb are the main elements that cause potential ecological risks in the study area, so they are selected as the main pollutants. Therefore, useful methods are urgently needed to prevent PTEs from inputting into the surrounding soil. At present, the effective technical measures mainly include: extraction and utilization of metals from tailings pond, and reduction of pollution based on advanced phytoremediation technology. Management measures should follow the principle that the polluter is responsible for pollution control.

It is worth mentioning that after investigation, it was found that few people in nearby villages got sick due to the tailings pond, and the villagers did not have any symptoms after eating the fruits of the fruit trees near the tailings pond. Therefore, it is preliminarily speculated that the villagers were not affected by the PTEs produced by the tailing pond. Moreover, we will study the health risk assessment of PTEs near the tailings ponds in-depth in the future.

### 3.6. Correlation between RI and Igeo and EF

**Figure 8:**
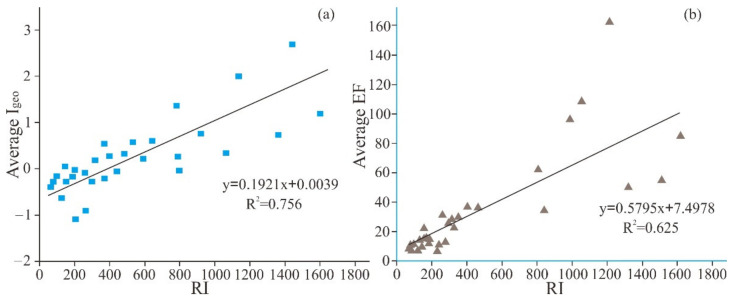
(**a**) Regression analysis between RI and Igeo; (**b**) Regression analysis between RI and EF.

Regression analysis was performed between the results of average RI and Igeo and EF. The positive correlation relationship between average RI and Igeo and EF is shown in Figure 7. The environmental pollution predicted by average RI corresponds roughly to Igeo and EF, and the correlation coefficients in Figure 8a,b are 0.756 and 0.625, respectively. This result is consistent with the previous author’s conclusion [51]. According to three quantitative risk indicators (RI, Igeo, and EF), the research shows that there is significant PTE pollution in the local area.

## 4. Conclusions

The study was performed to investigate the pollution characteristics and environmental risks of PTEs in the soil around the tailings pond. Therefore, the characteristics of the pollution sources of PTEs were revealed through multivariate statistical methods, and the potential ecological risks were assessed based on the PERI method. The soil pollution level is calculated quantitatively using Igeo and EF. The spatial distribution trend showed that the overall trend was: the closer to the tailing pond, the higher the content of PTEs. The results showed that the accumulation degree of PTEs in the soil samples is different. In addition, among these PTEs, the variations of Pb, Zn and Hg were the most significant. The temporary difference in the concentration of PTEs may be related to environmental factors and high-intensity mining and smelting activities. The results of multivariate statistical analysis suggested that among the existing sources, the accumulation of tailings ponds formed by mining and smelting activities is the dominant factor of emission, and amalgamation method mainly led to high Hg concentrations. The results of Igeo, EF and PERI showed that Sb, As, Pb, Cu and Hg were the main pollutants in the soil, which had significant potential ecological risks compared to other potentially toxic elements. Therefore, mine managers should reinforce the tailings pond to prevent the tailings from seeping downstream. It is proposed that after the cessation of gold mining and smelting activities, mine tailings should be properly handled. Moreover, if necessary, measures to eliminate tailings ponds should be implemented.

## Figures and Tables

**Table 1:** Standard of contamination degree by enrichment factor.

EF	Contamination Degree
<2	No or minimal pollution
2–5	Moderate pollution
5–20	Significant pollution
20–40	Strong pollution
>40	Extremely strong pollution

**Table 2:** Grading evaluation for potential ecological harm.

E_r_^i^	Grades of a Single Element of Ecological Risk	RI	Grades of a Number of Elements of Potential Ecological Risk
E_r_^i^ < 40	Slight pollution	RI < 150	Low
40 ≤ E_r_^i^ < 80	Moderate pollution	150 ≤ RI < 300	Moderate
80 ≤ E_r_^i^ < 160	Significant pollution	300 ≤ RI < 600	High
160 ≤ E_r_^i^ < 320	Strong pollution	RI ≥ 600	Serious
E_r_^i^ ≥ 320	Extremely strong pollution		

**Table 3:** Statistical analysis of PTE concentrations in soil samples near the tailing pond.

Elements	Sb	As	Cd	Cu	Pb	Zn	Hg
Minimum (mg/kg)	2.40	0.65	0.15	8.26	41.50	5.53	0.026
Maximum (mg/kg)	93.8	59.45	5.16	533.20	1875.28	449.36	5.28
Mean (mg/kg)	16.24	28.29	0.99	171.04	263.25	99.73	0.72
Standard deviation	12.07	15.99	1.41	173.34	348.91	84.59	1.35
Median	3.34	12.87	0.92	89.4	132	96	0.46
Variation coefficient (%)	74.32	56.52	142.42	101.34	132.54	90.25	187.51
Chinese soil quality Guideline ^a^	10	25	0.60	100	300	250	0.50
The background values ^b^	1.08	9.4	0.0534	23.1	24.7	97.2	0.0576

NA: Not available. ^a^: The Grade II (pH = 6.5–7.5) of the Chinese Soil Environment Quality Standard (GB15618-1995) was selected as the assessment standard. Grade II could be employed to protect human health as the critical value (Chinese Environmental Protection Administration (CEPA) 1995). ^b^: The background values for the PTEs in soils were taken, at the provincial level, from the report “The Background Concentrations of Soil Elements of China” (China National Environmental Monitoring Center 1990) [45].

**Table 4:** Pearson correlation matrix and component matrix for the elements’ concentrations in the surrounding soils.

Elements	Rotated Component Matrix		Pearson Correlation Matrix						
	PC1	PC2	Sb	As	Cd	Cu	Pb	Zn	Hg
Sb	**0.903**	−0.062	1.00	0.913 **	0.035	0.813 **	0.825 **	0.123	−0.037
As	**0.902**	−0.178		1.00	0.046	0.827 **	0.542 *	0.181	−0.168
Cd	−0.121	**0.864**			1.00	0.116	0.026	0.786 **	−0.128
Cu	**0.837**	−0.183				1.00	0.461 *	0.131	−0.174
Pb	**0.772**	0.116					1.00	0.073	−0.032
Zn	−0.06	**0.936**						1.00	0.069
Hg	**0.621**	**0.594**							1.00
Eigen values	4.07	1.08							
Cumulative percent (%)	58.1	78.5							

* Correlation is significant at *p* < 0.05 (2-tailed); ** correlation is significant at *p* < 0.01 (2-tailed). Bold black values represent that PTEs were strongly associated in the same factor with high loadings (>0.60).

**Table 5:** Potential ecological risk coefficient (E_r_^i^) and potential ecological hazard index (RI) of the studied elements in soils.

Elements	E_r_^i^							RI
	Sb	As	Cd	Cu	Pb	Zn	Hg	
Minimum	34.72	8.97	2.79	10.93	40.07	10.07	18.71	67.29
Maximum	139.72	262.76	39.29	61.76	64.01	34.01	1242.35	1612.55
Mean	66.18	61.65	25.86	43.55	47.43	17.43	205.60	333.53

## Data Availability

Not applicable.
